# Supplementary material for: Many-Body Contributions in Water Nanoclusters
Source: ACS Nano. 2023 Jan 25;17(3):1959–64. doi: 10.1021/acsnano.2c06077 (PMC10781035; doi:10.1021/acsnano.2c06077)
Supplement: Supplementary file 1 — nn2c06077_si_001.pdf [file nn2c06077_si_001.pdf]

Supporting Information for "Many-Body Contributions in Water Nano-Clusters"

David Abella,[†,‡] Giancarlo Franzese,[*,‡,¶] and Javier Hernández-Rojas[*,§]

†*Instituto de Física Interdisciplinar y Sistemas Complejos IFISC (CSIC-UIB), Campus UIB, 07122 Palma de Mallorca, Spain.*

‡*Secció de Física Estadística i Interdisciplinària - Departament de Física de la Matèria Condensada, Universitat de Barcelona, Martí i Franquès 1, 08028 Barcelona, Spain.*

¶*Institut de Nanociència i Nanotecnologia, Universitat de Barcelona, 08028 Barcelona, Spain.*

§*Departamento de Física e IUdEA, Universidad de La Laguna, 38205 La Laguna, Tenerife, Spain.*

E-mail: gfranzese@ub.edu; jhrojas@ull.edu.es

Results for the DC model

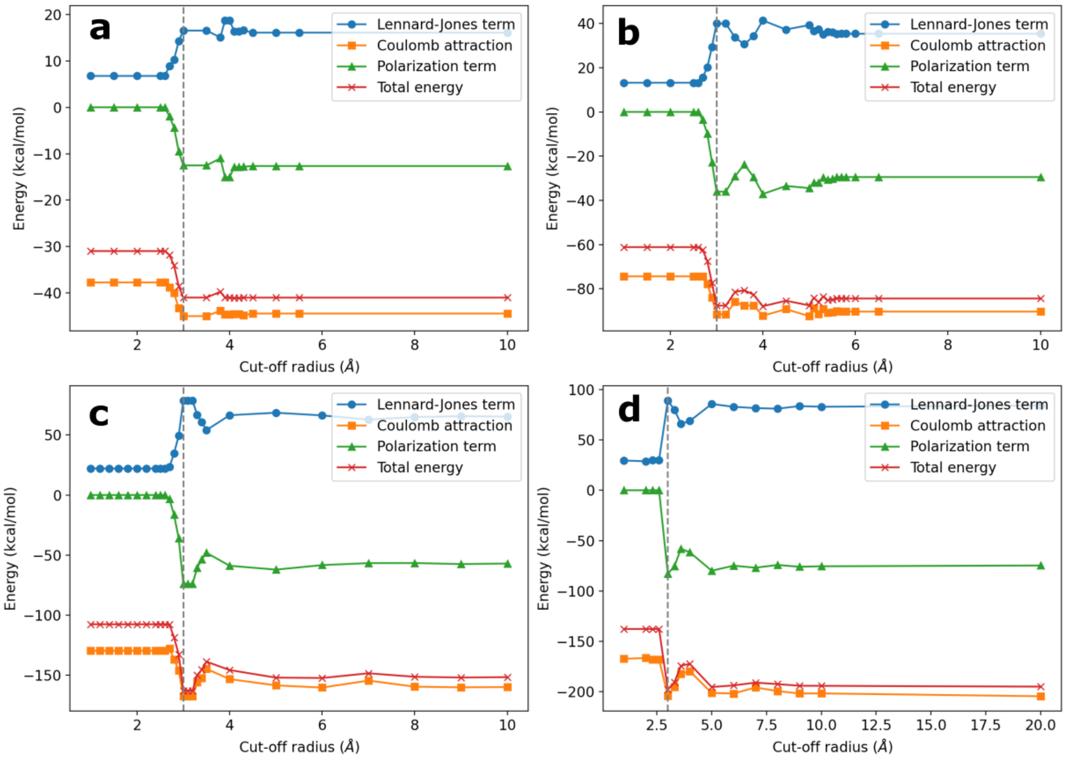

Figure 1: DC potential: As in Fig. 2 of the main text, but for water clusters of 6 (a), 10 (b), 16 (c), and 20 (d) molecules.

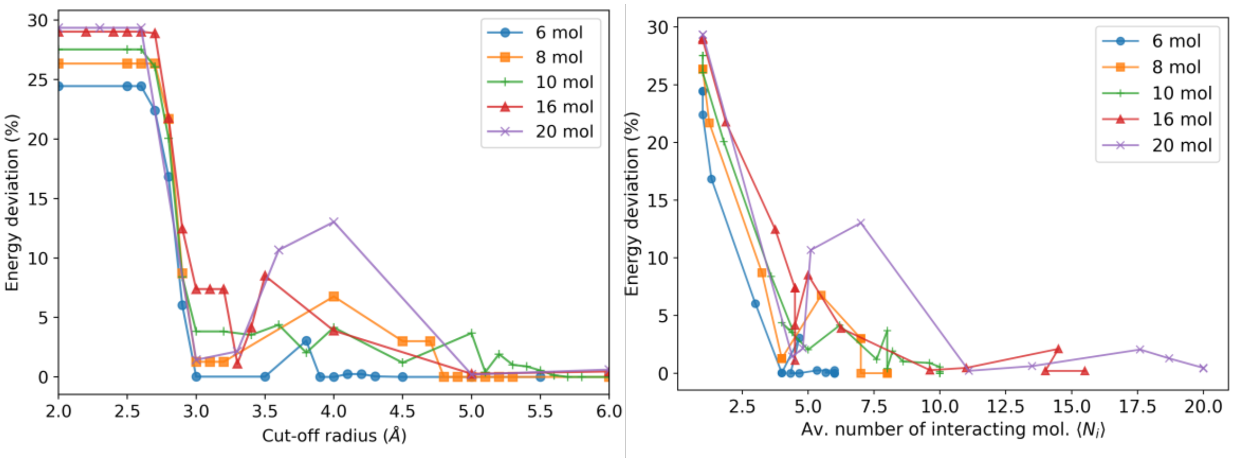

Figure 2: DC potential: Energy deviation from the reference DC value as a function of the cut-off radius r (left) and as a function of the average number of interacting molecules $\langle N_i \rangle$ (right) for clusters of 6 (blue circles), 8 (orange squares), 10 (green pluses), 16 (red triangles), and 20 (purple crosses) DC water molecules. The deviations are within 5% when the cut-off radius coincides with the first coordination shell ($r \sim 3$ Å). We take the absolute value of the energy deviations for the artificial minimum-energy configurations–at r between two consecutive coordination shells.

Minimum-energy configurations with the cut-off at the first coordination shell for the DC model

In Fig.3, we show that, when we include many-body effects until the first coordination shell, the minimum energy configurations are more similar to the results in the DC limit rather than the TIP4P-like limit.

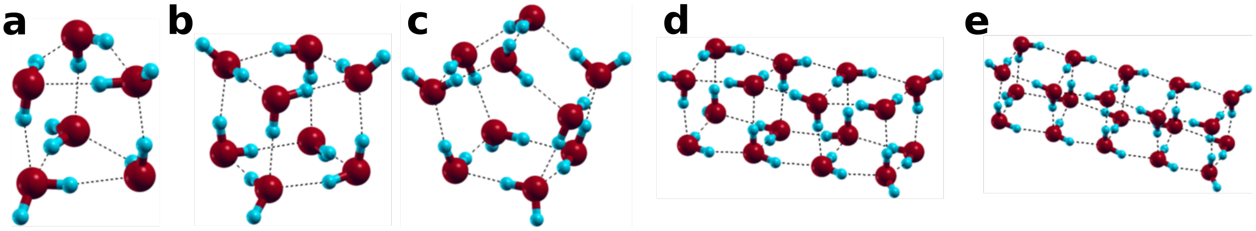

Figure 3: DC potential: The lowest-energy configuration for a cluster with 6 (a), 8 (b), 10 (c), 16 (d), and 20 (e) water molecules calculated for our model with the cut-off corresponding to the 1rst coordination shell ($r \sim 3$ Å).

Results for the MB-pol potential

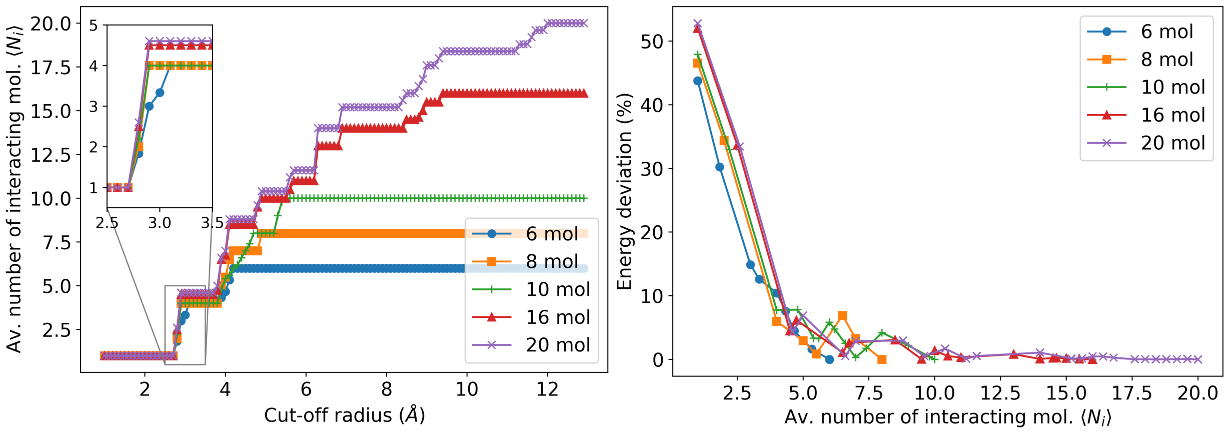

Figure 4: MB-pol potential: Average number of interacting molecules $\langle N_i \rangle$ as a function of the cut-off radius r (left) and energy deviation from the reference DC value as a function of $\langle N_i \rangle$ (right) for clusters of 6 (blue circles), 8 (orange squares), 10 (green pluses), 16 (red triangles), and 20 (purple crosses) MB-pol water molecules. The deviations are within 5% when the cut-off radius coincides with the first coordination shell ($r \sim 3$ Å). We take the absolute value of the energy deviations for the artificial minimum-energy configurations–at r between two consecutive coordination shells.

Among the available many-body models, MB-pol[1–3] has been shown to correctly perform the many-body analyses of small clusters.[4,5] Here we use the MBX calculator[6] to produce the results in Fig. 4. Due to the expensive computational cost, we adopt the minimum-energy configurations for the DC potential as a starting point for the MB-pol analysis.

Results for the KJ potential

The many-body KJ potential[7] is a rigid and four-site model similar to the DC potential. In both models, the polarizable site M is located on the bisector of the H-O-H bond angle. The most crucial difference between the two models is the sites associated with the dispersion force: the oxygen atom for the DC model and the M site for the KJ potential. Due to the expensive computational cost, we computed only two cluster sizes for the KJ potential: 8 and 20 molecules (Fig. 5).

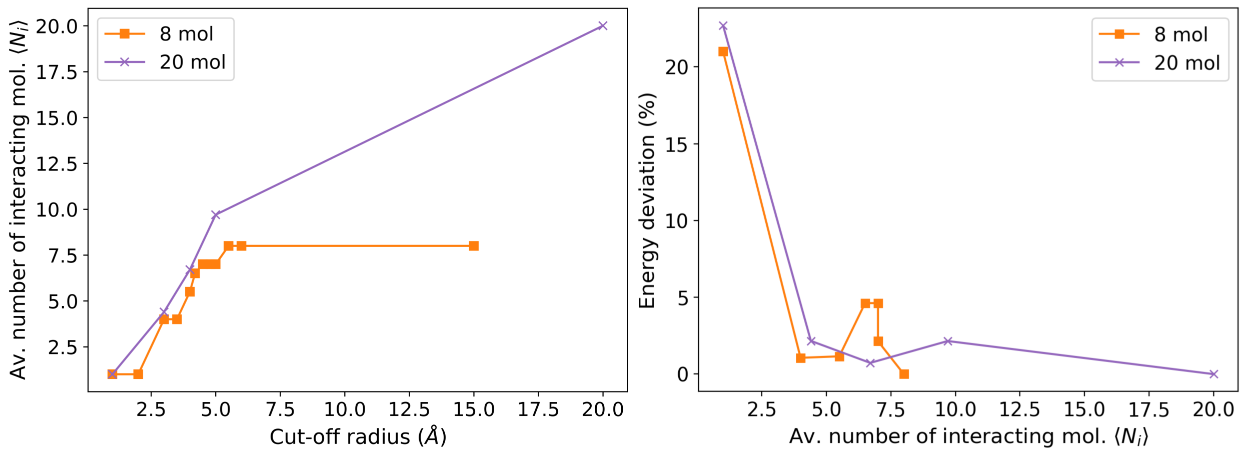

Figure 5: KJ potential: As in Fig. 4 but for the KJ water model for clusters of 8 (orange squares) and 20 (purple crosses).

References

(1) Babin, V.; Leforestier, C.; Paesani, F. Development of a "first principles" water potential with flexible monomers: Dimer potential energy surface, VRT spectrum, and second virial coefficient. *Journal of chemical theory and computation* **2013**, *9*, 5395–5403.

(2) Medders, G. R.; Babin, V.; Paesani, F. Development of a "first-principles" water potential with flexible monomers. III. Liquid phase properties. *Journal of chemical theory and computation* **2014**, *10*, 2906–2910.

(3) Babin, V.; Medders, G. R.; Paesani, F. Development of a "first principles" water potential with flexible monomers. II: Trimer potential energy surface, third virial coefficient, and small clusters. *Journal of chemical theory and computation* **2014**, *10*, 1599–1607.

(4) Medders, G. R.; Paesani, F. Infrared and Raman spectroscopy of liquid water through "first-principles" many-body molecular dynamics. *Journal of Chemical Theory and Computation* **2015**, *11*, 1145–1154.

(5) Reddy, S. K.; Straight, S. C.; Bajaj, P.; Huy Pham, C.; Riera, M.; Moberg, D. R.; Morales, M. A.; Knight, C.; Götz, A. W.; Paesani, F. On the accuracy of the MB-

pol many-body potential for water: Interaction energies, vibrational frequencies, and classical thermodynamic and dynamical properties from clusters to liquid water and ice. *The Journal of chemical physics* **2016**, *145*, 194504.

(6) MBX v0.7. `https://github.com/paesanilab/MBX`.

(7) Kozack, R. E.; Jordan, P. C. Polarizability effects in a four-charge model for water. *The Journal of Chemical Physics* **1992**, *96*, 3120–3130.
